# Disparities in fragility fracture and osteoporosis care in Africa

**DOI:** 10.1016/S2213-8587(24)00073-1

**Published:** 2024-03-22

**Authors:** Kate A Ward, Tafadzwa Madanhire, Kebba Marenah, Lisa K Micklesfield, Celia L Gregson

**Affiliations:** MRC Lifecourse Epidemiology Centre, University of Southampton, Southampton SO16 6YD, UK; MRC Unit The Gambia, London School of Hygiene and Tropical Medicine, Banjul, The Gambia; The Health Research Unit Zimbabwe, Biomedical Research and Training Institute, Harare, Zimbabwe; Department of Infectious Disease Epidemiology, Faculty of Epidemiology and Population Health, London School of Hygiene and Tropical Medicine, London, UK; Department of Orthopaedics and Trauma, Edward Francis Small Teaching Hospital, Banjul, The Gambia; SAMRC/Wits Developmental Pathways for Health Research Unit, School of Clinical Medicine, Faculty of Health Sciences, University of the Witwatersrand, Johannesburg, South Africa; The Health Research Unit Zimbabwe, Biomedical Research and Training Institute, Harare, Zimbabwe; Musculoskeletal Research Unit, University of Bristol, Bristol, UK

Over the coming decades, Africa is predicted to have the greatest increase in the number of older people worldwide. Older people in this region spend longer living with disability and dependence than do those in high-income settings, affecting individuals, families, communities, and health-care systems in some of the most resource-poor countries. In addition to shifting demographics, rapid urbanisation, double and triple burdens of malnutrition (the co-existence of two or three of overweight or obesity, undernutrition, and micronutrient deficiency), changing physical activity patterns and workplace environments, and climate change contribute to the growing prevalence of non-communicable diseases.^1^ Such diseases include cardiometabolic and musculoskeletal diseases, which often coexist as multimorbidity, along with communicable diseases such as HIV—an emerging chronic disease of ageing. Growing evidence shows that HIV and its treatment are important risk factors for fracture.^2^

In the Global Burden of Diseases, Injuries, and Risk Factors Study 2021, fragility fractures and osteoporosis (defined as low bone mineral density [BMD] and structural deterioration of bone) are categorised as other musculoskeletal disorders, yet their important contribution to the rising prevalence of injury-related and fracture-related disability, morbidity, and mortality is increasingly recognised.^3^ To date, a lack of awareness— and therefore health-care prioritisation—in Africa, plus a focus (led by high-income countries) on specialist techniques to assess BMD that are unavailable across much of Africa, has probably led to widespread underreporting of morbidity (eg, disability) and mortality associated with fragility fractures in the region, inevitably creating inequity in fracture prevention and care provision.^3^

Fractures of the proximal femur (hip) present a particular challenge to functional ability and survival. The incidence of hip fracture is projected to double in South Africa between 2020 and 2050—an increase that is expected to be seen across western, eastern, and southern African countries as populations continue to age and transition economically.^4,5^ In Black South African women and men, fracture outcomes are poorer than in Indian South Africans, with higher morbidity and mortality than seen in other countries worldwide.^6^ Harmonisation of cohorts across South Africa, The Gambia, and Zimbabwe showed that osteoporosis and osteopenia are just as common in ageing (≥40 years) Black African populations as in similarly aged US populations.^1^ Health-care systems, which are increasingly managing age-related multimorbidity, need to adapt to manage osteoporosis and prevent fragility fractures—for example, HIV services managing post-menopausal women should be routinely assessing fracture risk. Without such changes, health inequities will continue to grow for ageing populations in these settings.

Solutions lie in improving clinical awareness, primary care, and specialist training, and expanding access to potentially innovative diagnostic, treatment, and rehabilitation services. Traditionally, an osteoporosis diagnosis requires a dual-energy x-ray absorptiometry (DXA) scan confirming a BMD T score of –2·5 or lower (using internationally defined thresholds for diagnosis^1,7^). However, DXA scanners are expensive, require specialist support, and a reliable electricity supply. Additionally, there are few in number in Africa (for example, there are three in Zimbabwe, with a population of 15·2 million). Where such scanners are available, costs can be prohibitive to public health-care users.^8^ Additionally, as most people who sustain a fragility fracture have a femoral neck BMD T score greater than –2·5 (ie, not in the osteoporotic range), consideration of the many clinical risk factors besides BMD for fragility fracture risk is key. The widespread provision of DXA scanning is not practical in resource-constrained public health-care settings; as such, non-specialist fracture risk assessment tools should be a priority, with the only available tool in the region currently being FRAX. Validation of such tools will necessitate the collection of robust epidemiological data for fracture prevalence and incidence in different populations across Africa. Furthermore, evidence is scarce for the role of additional, context-specific clinical risk factors, such as HIV infection, which are likely to be important in addition to age, BMD, previous fracture, and alcohol intake.^9^ In many populations, data regarding parental fracture will be scarce owing to historically low life expectancy. Furthermore, research is needed to validate these new fracture risk assessment tools in African populations.

For more on **FRAX** see https://frax.shef.ac.uk/FRAX/

A further inequality arises from differences in provision within public and private health-care services. Access to medicines that are used commonly in high-income countries for the prevention of primary and secondary fractures is often possible only in private health-care systems in sub-Saharan Africa. This disparity largely reflects the lack of prioritisation of osteoporosis medicines, as they are not included on the WHO Essential Medicines list. Where private health-care plans exist in South Africa, osteoporosis is not considered a primary medical benefit; as such, there is no incentive to assess and treat fracture risk, although people with comprehensive medical insurance are reimbursed in the case of severe osteopenia, osteoporosis, and fracture.^8^ Medical pluralism is also common—particularly in west Africa, where traditional bone setters are usually the first point of contact on a complex care pathway—and can result in treatment delays. Equitable access to affordable treatments for osteoporosis and fractures should be a priority for health-care providers and policy makers.

Panel **Challenges and solutions to improving fragility fracture and osteoporosis care in Africa**

**Population-level challenges**

- Rapidly growing, ageing population in resource-poor settings
- Increase in non-communicable diseases, often leading to multimorbidity
- HIV as a chronic disease of ageing
- Rapid urbanisation
- Double and triple burdens of malnutrition
- Changing physical activity patterns and workplace environments
- Climate change

**Health-system challenges**

- Low levels of public and stakeholder awareness about bone health
- Historic focus on, and funding for, infectious diseases
- Historically isolated models of health care
- Multiple competing health-care priorities
- Poor access to specialist training and too few specialists in bone health
- Few validated tools for fracture risk assessment (only FRAX available, and in five sub-Saharan African countries (Botswana, Ethiopia, South Africa, Tunisia, and Zimbabwe)
- Poor access to dual-energy x-ray absorptiometry scanning services
- Medical pluralism

**Solutions**

- Co-design of public information resources to raise awareness
- Co-design of context-specific clinical guidelines
- Training and capacity strengthening
- Emerging fracture epidemiology and understanding of context-specific risk factors
- Validation of low-cost, scalable fracture risk assessment tools
- Consideration of osteoporosis treatments as essential medicines
- Integration of bone health in established pathways of health care (eg, HIV care)

The ultimate clinical manifestation of osteoporosis is a fragility fracture. The few studies conducted in Africa have been underpowered to estimate the prevalence or incidence of fractures. Except for South Africa, there remains a paucity of robust epidemiological data from Africa to evidence the increase in fragility fractures and to identify context-specific clinical risk factors. Notably, of 131 fracture liaison services surveyed globally in 2020, only one (in South Africa) was operational across western, eastern, and southern Africa.^10^

In conclusion, ageing populations in Africa do not have equitable access to diagnostic and treatment options to reduce the risk of fragility fractures and subsequent disability. Given the predicted exponential rise in demand placed by osteoporosis and fragility fractures on already stretched health-care systems, this matter requires attention (panel). Awareness is certainly increasing, with recognition of the importance of appropriate diagnostic and management pathways. Consulting communities and stakeholders will be important to ensure that practical, context-specific solutions are implemented. Providing equitable access to diagnostic services, creating implementable tools for diagnosis and treatment monitoring, and building capacity to provide specific expertise in osteoporosis care should be key goals for health-care services, policy makers, and governments.

## References

[1] Ward KA, Pearse CM, Madanhire T (2023). Disparities in the prevalence of osteoporosis and osteopenia in men and women living in sub-Saharan Africa, the UK, and the USA. Curr Osteoporos Rep.

[2] Hamill MM, Pettifor JM, Ward KA, Norris SA, Prentice A (2017). Changes in bone mineral density, body composition, vitamin D status, and mineral metabolism in urban HIV-positive South African women over 12 months. J Bone Miner Res.

[3] Gill TK, Mittinty MM, March LM (2023). Global, regional, and national burden of other musculoskeletal disorders, 1990–2020, and projections to 2050: a systematic analysis of the Global Burden of Disease Study 2021. Lancet Rheumatol.

[4] Odén A, McCloskey EV, Kanis JA, Harvey NC, Johansson H (2015). Burden of high fracture probability worldwide: secular increases 2010–2040. Osteoporos Int.

[5] Hawley S, Dela S, Burton A, Paruk F, Cassim B, Gregson CL (2022). Incidence and number of fragility fractures of the hip in South Africa: estimated projections from 2020 to 2050. Osteoporos Int.

[6] Paruk F, Matthews G, Gregson CL, Cassim B (2020). Hip fractures in South Africa: mortality outcomes over 12 months post-fracture. Arch Osteoporos.

[7] Kanis JA, McCloskey EV, Johansson H, Cooper C, Rizzoli R, Reginster JY (2013). European guidance for the diagnosis and management of osteoporosis in postmenopausal women. Osteoporos Int.

[8] International Osteoporosis Foundation (2011). The Middle East and Africa regional audit: epidemiology, costs & burden of osteoporosis in 2011.

[9] Gregson CL, Cassim B, Micklesfield LK, Lukhele M, Ferrand RA, Ward KA (2019). Fragility fractures in sub-Saharan Africa: time to break the myth. Lancet Glob Health.

[10] Clynes MA, Westbury LD, Dennison EM (2020). Bone densitometry worldwide: a global survey by the ISCD and IOF. Osteoporos Int.

